# Removable vs Fixed Bridge Work

**Published:** 1899-05

**Authors:** T. E. Turner

**Affiliations:** St. Louis, Mo.


ARTICLE VII.

### REMOVABLE VS. FIXED BRIDGE WORK.

T. E. TURNER, D. D. S., ST. LOUIS, MO.

Read before the St. Louis Dental Society, Dec. 6, 1898.

This is an age of progress and dentistry in common with many other sciences has kept well to the front; this is particularly so of dentistry in that branch of prosthesis known as crown and bridge work, upon the advent of which it was hailed as the great desideration, the zenith in the replacement of lost dental organs was thought to have been reached; teeth without plates became a reality, and fad or hobby with many practitioners. Its limitations were not fully comprehended, its principles were not understood, still it was made to serve in all manner and conditions of cases; a few scattering roots or teeth sufficed for the replacement of a full denture.

No doubt enthusiasm for the new method led many conscientious practitioners to make grave errors of judgment as to its availability in many cases, while the unscrupulous make use of it in every case where the fee is forthcoming. Such practice must and is having its legitimate fruitage in the many failures we see to-day—and they are many—while the future promises to be still more prolific. Bridge work must be used with discrimination only after a thorough understanding of the conditions involved; the prognosis should be extremely favorable. But a reaction has begun, and bridge work is held in disfavor with many practitioners, while others condemn it entirely. Thoughtful conservative dentists are coming more and more to realize that this method has only a limited scope, which cannot be exceeded without limiting the duration of the work, and are using it less and less.

Such is the usual history of all things new; new methods, new appliances, etc., are introduced as panaceas for all failures and diseases; great things are promised, great results expected; they are greeted with enthusiasm; many forgetting to "hold fast to that which is proven," forsake the tried and known for the untried and unknown, after being tried they eventually fall into their proper sphere and are recognized as valuable additions to their respective departments, in case they prove to be so.

Bridge work thus in time will take its proper place as a method for the replacement of lost teeth; its limitations will be more fully understood and respected (by reputable practitioners at least); a few scattered teeth or roots will no longer be used to carry a full denture, it being contrary to true mechanical principle; nor will teeth affected with pyorrhœa be used as abutments, but in its proper sphere will be recognized as a legitimate operation, and the best method yet devised for the purpose for which it was intended, provided it be skillfully constructed in accordance with true mechanical principles; and yet, however skillfully constructed or properly placed, the result falls far short of the ideal. Certatn defects are inherent in fixed bridge work, and cannot be eliminated, however well the piece may be constructed; it may conform to true mechanical principles, be correct from an æsthetic point of view and be within its proper sphere, yet it is a failure in some measure, for in the very nature of things it cannot fulfill all the requirements of an ideal bridge. In the above reference to bridge work, the ordinary is meant, one continuous piece terminating with a crown at each end, the fixed or immovable bridge.

Some of the most prominent defects of this class of work are these: First. The difficulty of making repairs. When it becomes necessary to replace a broken facing, the result is usually unsatisfactory, if attempted while the piece is in position. If the bridge is to be taken off, the crown must be cut open, bent and twisted out of shape. In many cases they are utterly ruined, as far as fitting again is concerned, but are generally patched and used again. This is often made necessary by the fact that a sufficient fee cannot be commanded, except in few instances, which would justify the dentist in making a new crown for the abutements, besides replacing the broken facing; he, therefore, does not feel called upon to do more than is absolutely necessary. If it becomes necessary to repeat this operation a second or third time, the condition of the crown may be easily imagined. They will be composed in most part of solder, and are rigid, unyielding and ill fitting, but still made to do service.

I am sure this very thing accounts for many of the poorly fitting, disreputable looking bridges we often see. We judge them in their present condition, forgetting that possibly they may have got in such condition through a series of repairs. And then some bridges have a faculty of getting loose at one end only, then it becomes necessary to sacrifice the crown that has proven true.

Second. The so-called self-cleaning space is a misnomer, a delusion, and a most convenient place for the lodgment of food. It is almost, if not impossible to keep them clean. It may be possible, but I have yet to see one kept so. I think a bridge with these spaces is not so cleanly as a saddle bridge, but it possesses this advantage —you can look under it and see what is there. In some cases these self-cleaning spaces interfere very seriously with speech and are a great annoyance to the tongue, while for all the vile odors imaginable I have never found the equal of a recently extracted fixed bridge.

Third. In many cases the teeth to be used as abutments diverge from the parallel, necessitating no undue amounr of grinding or a stretching and straining of the piece until the fit of the bands is destroyed.

Fourth. The fixed bridge as usually constructed fails to restore the full contour of the lost tissues; this, of course, would not apply to saddle bridges, as the objections under the first three do.

That the fixed bridge does not fully meet the requirements is demonstrated by the continual striving for something better, in the various forms of removable pieces that are suggested and introduced from time to time, thus overcoming in most cases and in great part the objections to the immovable work.

The ideal bridge must belong to the removable class, as it alone can possess all the requisites necessarily inherent in an ideal bridge, and must conform to the following requirements: The lost tissues, teeth, gums and process must be exactly restored, it must not be too complicated of construction (which is a serious objection with some system); must be easily removed and replaced by the wearer and yet must be perfectly rigid and immovable when in position; the teeth that are supplied should be attached to a small plate or saddle resting firmly on the gums, thus relieving the piers of any undue strain or pressure, they being used principally to hold the bridge in position. A bridge filling the above requirements can be easily repaired and can be kept clean, the two most essential requirements.

It is not intended to condemn fixed bridge work in toto, for I am sure that in many cases it would be indicated in preference to removable work, in the present state of developments of the removable systems; but my desire is to call your attention to the limitations and possibilities of each system, so that with these well in mind we may at least form a correct opinion, as the cases are presented to us. It must be admitted that fixed or immovable bridge work has attained its highest perfection, and any further progress must of necessity be along the line of removable work.

Bridge work should only be inserted after thorough study of the case in hand, the conditions to be met should be fully appreciated, discrimination should be made between the fixed and removable systems, with the difficulties to be overcome and the mechanical principles involved well in mind; it is well also, in this connection. to keep in mind the fact that partial gold plates, made stable if necessary with clasps on gold crowns, are indicated in many cases instead of either form of bridge work.

There is probably less objection to fixed bridge work in the incisor region than in the molar and bicuspid, because the contour of the lost teeth is more nearly restored, it is possible to keep them cleaner, and facings can be more easily replaced while the piece is in the . outh.

In this connection a brief mention of some of the crowns and attachments used in removable work would probably not be amiss. Probably the best crown for the six anterior teeth is the Richmond removable crown with a split pin, by which the crown can be made tight again if it should become loose. If this crown is properly made, great care being given to the minute details, I am sure it will do all that is expected of it, being easily removed, yet perfectly rigid when in place. For the molars and bicuspids, the telescoping crowns are among the best forms. They must be accurately made, and the crown should be of some length, not too short.

One of the most admirable systems yet devised is described by Dr. Rhein in the *Cosmos* for February, 1894. This overcomes every objection that can be made to fixed bridge work. Mutilation of the teeth is reduced to the minimum, it being only necessary to shorten the articulating surfaces, split crowns being used if the sides are not parallel, the edges being drawn together after the crown is in place, which requires a high degree of skill, as do also the other numerous details of the system, to accomplish the desired result, together with an infinite amount of patience and time, and a good fee in view—a combination rarely met with.

Various forms of clasps are also used as a means of holding in position removable bridge work. These pieces border very closely on plate work, in fact, may be said to be the connecting link between the true bridge and the plate, but are probably of doubtful value.

I have also used the Condit attachments in several cases. In one, two bicuspids were supplied, being attached with rubber to a small gold plate or saddle, which rested on the bridge, crowns being placed on the cuspid and molar, with the Condit attachments soldered on; but I am awaiting developments before using them further.

While all these methods are useful and valuable in many cases, I cannot but believe that the future has in store something better—system that will be easily constructed, durable, positive and universal.—*Dental Review*.

